# Comparison of 16 mm OSU‐Nag and COMS eye plaques

**DOI:** 10.1120/jacmp.v13i3.3632

**Published:** 2012-05-10

**Authors:** Hualin Zhang, Frederick Davidorf, Yujin Qi

**Affiliations:** ^1^ Department of Radiation Oncology Indiana University School of Medicine Indianapolis IN 46202; ^2^ Department of Ophthalmology The Ohio State University Columbus OH 43210; ^3^ Shanghai Institute of Applied Physics Chinese Academy of Science Shanghai 201800 China

**Keywords:** brachytherapy, COMS eye plaque, OSU‐NAG eye plaque, ${\;}^{125}I$, ${\;}^{131}\text{Cs}$

## Abstract

OSU‐NAG eye plaques use fewer sources than COMS‐plaques of comparable size, and do not employ a Silastic seed carrier insert. Monte Carlo modeling was used to calculate 3D dose distributions for a 16 mm OSU‐NAG eye plaque and a 16 mm COMS eye plaque loaded with either Iodine‐125 or Cesium‐131 brachytherapy sources. The OSU‐NAG eye plaque was loaded with eight sources forming two squares, whereas the COMS eye plaque was loaded with thirteen sources approximating three isocentric circles. A spherical eyeball 24.6 mm in diameter and an ellipsoid‐like tumor 6 mm in height and 12 mm in the major and minor axes were used to evaluate the doses delivered. To establish a fair comparison, a water seed carrier was used instead of the Silastic seed carrier designed for the traditional COMS eye plaque. Calculations were performed on the dose distributions along the eye plaque axis and the DVHs of the tumor, as well as the 3D distribution. Our results indicated that, to achieve a prescription dose of 85 Gy at 6 mm from the inner sclera edge for a six‐day treatment, the OSU‐NAG eye plaque will need 6.16 U/source and 6.82 U/source for ${\;}^{125}I$ and ${\;}^{131}\text{Cs}$, respectively. The COMS eye plaque will require 4.02 U/source and 4.43 U/source for the same source types. The dose profiles of the two types of eye plaques on their central axes are within 9% difference for all applicable distances. The OSU‐NAG plaque delivers about 10% and 12% more dose than the COMS for ${\;}^{125}I$ and ${\;}^{131}\text{Cs}$ sources, respectively, at the inner sclera edge, but 6% and 3% less dose at the opposite retina. The DVHs of the tumor for two types of plaques were within 6% difference. In conclusion, the dosimetric quality of the OSU‐NAG eye plaque used in eye plaque brachytherapy is comparable to the COMS eye plaque.

PACS number: 87.56B, 87.55k, 87.55kh

## I. INTRODUCTION

Eye plaque brachytherapy is a well‐recognized treatment technique for the management of ocular melanomas. According to clinical reports, eye plaque brachytherapy has a similar survival rate compared to surgical enucleation for medium size uveal tumors.^(^ ^1^ ^–^ ^3^ ^)^ The Collaborative Ocular Melanoma Study (COMS) eye plaque has been popular for decades for its standardization on plaque dimensions and number of seeds. Many studies have been published which investigate the pros and cons of the COMS eye plaque, although other types of eye plaques are still available and used at various centers.^(^ ^4^ ^–^ ^8^ ^)^ In the conventional COMS eye plaque brachytherapy, heterogeneity was introduced by both the gold plaque itself and the Silastic seed carrier (Dow Corning, Midland,MI). Chiu‐Tsao et al.^(^ ^5^ ^)^ first studied the impact of the Silastic carrier and the gold plaque using Monte Carlo methods and thermoluminescent dosimeters (TLD). TLD measurements were also performed by de la Zerda et al.^(^ ^6^ ^)^ and similar results were confirmed. These studies showed that the Silastic carrier and the gold plaque could reduce dose by up to 10% at a distance of 10 mm on the plaque axis compared with water. The American Brachytherapy Society published its recommendations regarding plaque radiotherapy in 2003.^(^ ^9^ ^)^ Among other literature, in 2005 Astrahan et al.^(^ ^10^ ^)^ reported that for ${\;}^{125}I$ sources, use of the Silastic carrier results in a dose reduction of at least 10% in the vicinity of the tumor apex where the dose is usually prescribed, compared to absorbed dose to water, and greater than 10% at the tumor base and adjacent retina where the dose is closely watched. In another study, Astrahan et al.^(^ ^4^ ^)^ suggested that the Silastic seed carrier insert has caused a list of concerns, because it not only reduces doses at the points of interest, but also creates a set of additional difficulties, such as handling Silastic insert by forceps behind an L block, and disassembling and sterilization of the eye plaque set. According to Astrahan, a water equivalent seed carrier, or just water, is dosimetrically desirable; but a thin, gold alloy, seed guide insert would make the job of handling and sterilizing eye plaque assembly easier if the insert must be used. Recently, Melhus and Rivard^(^ ^11^ ^)^ studied COMS eye plaques dose reduction due to the gold alloy and Silastic insert using Monte Carlo technique, and published the correction functions for using 6711 ${\;}^{125}I$, model 200 ${\;}^{103}\text{Pd}$ and Cs‐1 ${\;}^{131}\text{Cs}$ sources separately. Thomson et al.^(^ ^12^ ^)^ investigated COMS eye plaque with Monte Carlo simulation on its gold plaque intersource effect, as well as the air interface and bone effect. Very recently, Zhang et al.^(^ ^13^ ^)^ studied a COMS eye plaque for its tumor and eyeball dose volume histograms when using either ${\;}^{125}I$ or ${\;}^{131}\text{Cs}$ sources. Zhang and colleagues also verified the accuracy of TG43U1 algorithm and intersource effect through a set of Monte Carlo simulations. In another recently published study, Rivard et al.^(^ ^14^ ^)^ compared the dose calculation methods used in COMS eye plaque calculations. All of the above studies have added new understanding and knowledge of the COMS eye plaque.

**Figure 1:**
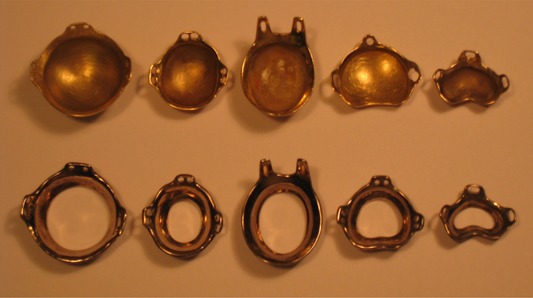
Several typical designs of the OSU‐NAG eye plaques. The upper panel is the OSU‐NAG eye plaques used in treatments; the lower panel is the corresponding dummy plaque used for determining the suturing location during the procedure.

The OSU‐NAG eye plaque was developed in our clinic and has been used since the early 1980s to treat uveal melanomas. Figure 1 shows the various designs for the OSU‐NAG eye plaques.^(^ ^8^ ^)^ The OSU‐NAG eye plaque does not use a Silastic seed carrier, but directly glues sources onto its concave surface at conveniently determined and equally spaced locations. For tumors with a diameter of 6 mm or less, four sources were used; 6 or 8 sources were used for tumors less than 10 mm but greater than 6 mm, depending on ellipsoidal or circular shapes. More sources could be used for larger tumors and plaques. Each time the eye plaque was assembled, these locations were measured by ruler and marked by pen before the sources were glued. A 1 mm offset was used to accommodate the curved surface of the plaque when two or more circles of sources were used. Compared with COMS eye plaques, the OSU‐NAG eye plaques have shown some advantages. From the clinical perspective, intraoperatively, the larger eyelets in the OSU‐NAG design are easier to suture to the sclera than the smaller eyelets in the COMS plaque. The OSU‐NAG plaque also can be designed and manufactured to treat tumors of nearly any shape. For oval shaped tumors, we use oval shaped plaques. For round shaped tumors, the round plaques are used. Notched plaques are used for very anterior tumors or peripapillary tumors to minimize dose to the optical nerve.^(^ ^15^ ^)^ Posterior uveal melanomas are treated with a plaque which has “rabbit ears”. This rabbit ear plaque is easier to secure to the globe than the COMS plaque for the posteriorly located tumors.

From a radiation physics perspective, the OSU‐NAG eye plaque is also advantageous over the COMS plaque. In the OSU‐NAG eye plaque, the brachytherapy sources are directly glued to the plaques onto the predetermined positions, allowing a more accurate dose calculation than the COMS, which uses the Silastic carrier on a concave plaque.^(^ ^5^ ^,^ ^16^ ^)^ Most planning systems have not developed a means for accommodating the dose reduction at locations of interest. Because the Silastic seed carrier is made of soft rubber material, when the sources are put in the grooves, their locations cannot always be exactly fixed each time, though on most occasions the variation is not significant. It should be noted that the OSU‐NAG eye plaque also has source positioning errors because the source locations were determined and measured by users in every case; however, since it uses fewer sources at simple coordinates, the real positions of sources could be easily measured again. Another issue is the sterilization of eye plaque sets. Our initial experience with using COMS eye plaques indicated that Silastic seed carrier makes sterilization difficult, as also reported by Astrahan et al.^(^ ^10^ ^)^ A potential disadvantage to reducing the number of sources compared to those used in a standard COMS eye plaque is an increase in dose heterogeneity. It should be noted that the modern trend in radiation therapy (e.g., IMRT and radiosurgery) is to use more sources of radiation in which each irradiates a sharply collimated portion of the tumor in order to achieve homogeneous and highly conformal dose distributions with a steep gradient outside the target volume. However, if using fewer sources does not impair the dose conformality, this would be clinically advantageous.

This study evaluated the dosimetric quality of the OSU‐NAG plaque in treating a sample eye tumor using both the ${\;}^{125}I$ and ${\;}^{131}\text{Cs}$ sources compared with the 16 mm COMS eye plaque. We use ${\;}^{131}\text{Cs}$ source in this study simply because this source is becoming more popular for brachytherapy applications, although its clinical use for eye plaques has not been reported.

## II. MATERIALS AND METHODS

### A. COMS eye plaque

The COMS eye plaque is currently available in seven standard sizes with diameters ranging from 10 mm to 22 mm in 2 mm increments. Its body is a portion of spherical shell 0.5 mm thick with a cylindrical collimating rim.^(^ ^12^ ^)^ The gold alloy used in the COMS plaque is 77% gold, 14% silver, 8% copper, and 1% palladium by weight. The brachytherapy sources are loaded in molded troughs in the Silastic source carrier insert that fits snugly in the concave aspect of the plaque. A 16 mm COMS plaque is used in this study. Thirteen sources of equal source strength were placed to treat ocular tumors ranging from 10 to 12 mm in diameter, in compliance with the COMS's recommendation that a tumor‐free margin of 2 to 3 mm should be maintained. The coordinates of 13 sources in a 16 mm COMS eye plaque, as described by Thompson et al.,^(^ ^12^ ^)^ were used in the calculations. A water‐source carrier was chosen in our study to achieve a better dose distribution for the implant, as suggested by Astrahan,^(^ ^4^ ^)^ and also to have a fair comparison with the OSU‐NAG eye plaque, which does not have a Silastic source carrier. A detailed description of the COMS eye plaques can be found in the literature.^(^ ^12^ ^,^ ^14^ ^,^ ^15^ ^)^

### B. OSU‐NAG eye plaque

**Figure 2:**
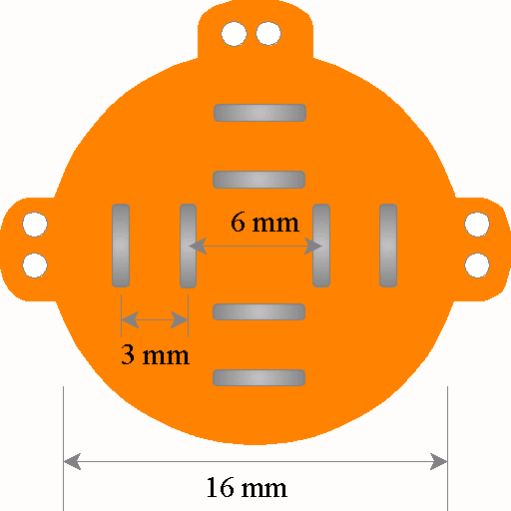
Schematic diagram of the OSU‐NAG eye plaque used in this study.

The OSU‐NAG plaque is a portion of spherical shell 0.5 mm thick with ellipsoidal or cylindrical collimating rim, which would allow users to conveniently select a pertinent shape for a particular tumor. The gold alloy used in the OSU‐NAG plaque is the same as that used in the COMS eye plaques. Figure 2 shows the 16 mm cylindrical OSU‐NAG plaque and positions of sources used in this study. Unlike the 16 mm COMS eye plaque loaded with thirteen sources approximating three isocentric circles, the OSU‐NAG eye plaque of this study used only eight sources forming two concentric squares at the easily determined locations. The seeds' locations were measured and marked on the surface of plaque before the sources were glued in place. After the plaque is assembled, a second physicist checks the coordinates of the seeds using a caliber ruler, and if a difference greater than 1 mm is found, enters the real distances into the treatment planning system (BrachyVision, Varian Medical, Alto Palo, CA).

### C. IsoAid IAI‐125A ${\;}^{125}I$ source

The IsoAid model IAI‐125A Iodine‐125 source, manufactured by IsoAid (IsoAid LLC, Port Richey, FL) is 4.5 mm in length, with a 0.8 mm outer diameter. The ${\;}^{125}I$ radionuclide is uniformly coated on the surface of a 3 mm long and 0.5 mm diameter silver rod, which also serves as an X‐ray marker. The entire assembly is encapsulated in a 0.05 mm thick titanium tube. The dosimetric characteristics of a single source were determined using experimental and theoretical methods,^(^ ^17^ ^)^ and its application in COMS eye plaque has also been theoretically studied by our group.^(^ ^13^ ^)^

### D. Isoray Cs‐1 ${\;}^{131}\text{Cs}$ source

The IsoRay Cesium‐131 brachytherapy source (Model Cs‐1 Rev2; IsoRay Medical Inc., Richland, WA) has a physical length of 4.5 mm with 0.1 mm thick caps on each end and an outer dimension of 0.8 mm. The source was manufactured by encapsulating an inner core with a 0.05 mm thick titanium wall. The center X‐ray marker is a 0.25 mm diameter gold wire. The wire is coated by a glass and ceramic material. The outside diameter of the source inner core is 0.65 mm. The active length of the source is 4.0 mm. The dosimetric behavior of a single source has been recently investigated by several groups including ours.^(^ ^18^ ^–^ ^20^ ^)^ One of our studies also investigated the dosimetric property of multiple Cs‐1 sources in COMS eye plaque brachytherapy,^(^ ^13^ ^)^ as did other groups.^(^ ^11^ ^)^

### E. Tumor and eyeball

**Figure 3:**
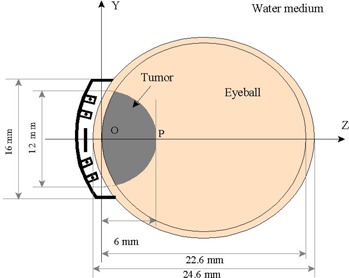
Schematic diagram of the eye plaque, an eye ball, a sample eye melanoma tumor, and the coordinate system used in the Monte Carlo simulations for this study.

This study used a 16 mm COMS and a 16 mm OSU‐NAG eye plaque separately to treat a tumor which is a part of ellipsoidal volume with a diameter in the major and minor axis of 12 mm and a height of 6 mm. (Fig. 3). The origin of the coordinates system is located at the inner sclera edge (Z = 0). Tumors of this shape and size are commonly seen at the Ohio State University ophthalmology cancer clinic. The diameter of the eyeball was assumed to be 24.6 mm.

### F. Monte Carlo (MC) simulations

A Monte Carlo N‐particle Transport Code (MCNPX, Version 2.5)^(^ ^21^ ^)^ was used to calculate the doses in water around a 16 mm COMS eye plaque loaded with either ${\;}^{125}I$ or ${\;}^{131}\text{Cs}$ sources. The sources of IAI‐125A ${\;}^{125}I$ and Cs‐1 ${\;}^{131}\text{Cs}$ were benchmarked by Monte Carlo simulations in our previous studies^(^ ^13^ ^,^ ^17^ ^,^ ^20^ ^)^ with MCNP5 and MCNP4C. We further verified that the results of MCNPX agree with our previous data from MCNP5 and MCNP4c within 1%. There are several tally types available in this code for dose calculation. The *F4 calculates the average photon energy fluence over a tally cell in ${\text{MeV}/\text{cm}}^{2}/\text{photon}$, which is then converted to the dose by incorporating the updated mass energy absorption coefficients (${\text{cm}}^{2}/g$).^(^ ^22^ ^)^ We used this tally type in all of our dose simulations.

The photon interaction cross‐section file used in this study was the DLC‐200 library distributed by the Radiation Shielding Information Computing Center (RSICC).^(^ ^23^ ^)^

In order to relate the calculated dose rate to the source strength, the Monte Carlo calculated dose rate value was converted to dose rate per air kerma (U) using Eqs. (1) and (2):

(1) $$\dot{D}\;(MeV/(g\cdot dis)=D\prime (\frac{MeV}{g\cdot \gamma })\cdot f\cdot (\frac{\gamma }{dis})$$

where, *D′* is kerma per photon γ, with the unit of MeV/(g.γ), and *f* is the ratio of photon/disintegration, which is 1.476 for ${\;}^{125}I$ and 0.729 for ${\;}^{131}\text{Cs}$ source.^(^ ^24^ ^)^ The conversion factor for MeV/(g.dis) to Gy/(h.U) was derived as follows and was directly incorporated into the MCNPX code in order to obtain the dose rate in the desired units:

(2) $$1\frac{MeV}{g\cdot dis}=21.34*c\;(Gy/h\cdot U)$$

where, *c* is a conversion factor between the air kerma strength U and contained activity mCi, namely 1 U=c mCi (contained). *c* is determined from the air kerma strength Monte Carlo simulation of a single source.

It should be noted that the air kerma strength of a single source in void space with dry air tally cells was first simulated for each source model before the simulation of the eye plaque implant in water, and the activity c (in mCi) to ensure that 1 U source strength was determined. This activity *c* was then incorporated into the simulations of the eye plaque implant in water. In this way, we eliminated the uncertainties in determining the ratio of apparent/content activity, which is hard to determine accurately, and made certain that the calculated dose was in the unit of Gy/U. Two separate simulations in void space confirmed that *c* was 1.375 for IAI‐125A and 2.546 for Cs‐1.

In all eye plaque implant calculations, the eye plaque was virtually placed at the center of spherical water phantom 15 cm in radius. This phantom size is also used in determining TG43U1 parameters of a single source of each model. The real size of the eye and surrounding tissue is smaller than the phantom used in this study. The purpose of using such a big phantom is merely to make our TG43U1 parameters comparable with published data. Therefore, the data generated from this study can be used to compare with the results obtained from the clinical treatment planning systems.

In order to calculate the DVHs, the spherical tally cells measuring 1 mm in diameter were placed uniformly across the tumor and eyeball volumes at 1 mm intervals to score the doses. These spherical tally cells were designed not to overlap each other to ensure the events would not be counted twice. The coordinates of the tally cells were determined and output to the MCNPX input files by a FORTRAN code designed by an author (HZ). The scored doses were then analyzed using Origin graphical software (OriginLab Corporation, Northampton, MA) to output DVH values. The energy spectrum of ${\;}^{125}I$ was taken from the TG43U1 report.^(^ ^25^ ^)^ The spectrum of the ${\;}^{131}\text{Cs}$ source was taken from the NNDC report.^(^ ^24^ ^)^

In all simulations, a cutoff energy of 5 keV was used for both the photon and electron energy in order to replicate the NIST's source calibration condition using the Wide Angle Free Air Chamber (WAFAC) detector and also to comply with the TG43U1 recommendation.

A history number of $2\;\times \;10^{8}$ was used in each simulation. This resulted in the statistical uncertainty for air kerma strength less than 0.1%, and for the eye plaque implant dose less than 1% at the distance on the central axis from the inner sclera edge within 20 mm. When the distance is within 30 mm, the overall statistical uncertainty for the implant dose is below 1.5%.

## III. RESULTS

### A. Single source data

**Figure 4:**
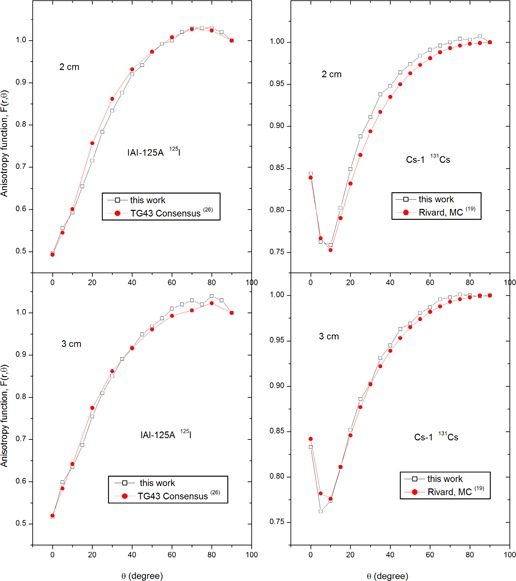
A comparison of the anisotropy functions between this study and published data.

Prior to the simulations of multiple source implants, the data of a single source of either IAI‐125A or Cs‐1 were generated. The results were in good agreement with the published data.^(^ ^17^ ^,^ ^19^ ^)^ Tables 1 and 2 give the radial dose functions of IAI‐125A and Cs‐1 in a water phantom, compared with the published data. The dose rate constant for I‐125 source was 0.979±3% cGy/hr/U (compared to 0.982 cGy/hr/U by Meigooni et al.,^(^ ^17^ ^)^), Cs‐131 was 1.059±3% cGy/hr/U (compared to 1.059 cGy/hr/U by Wang and Zhang^(^ ^20^ ^)^). The anisotropy functions were also found to be in good agreement with published data (Fig. 4).^(^ ^20^ ^,^ ^26^ ^)^

**Table 1:** IAI‐125A radial dose functions obtained by this work compared with a previous study by Meigoonie et al.^(^ ^17^ ^)^ in a water phantom.

	*g(r)*	*g(r)*	*Ratio*
*r (cm)*	*A. Meigooni et al.* ^(^ ^17^ ^)^ *(PTRAN)*	*B. This work, 30 cm in diameter spherical water phantom, torus tally cells. (MCNPX)*	*(A/B)*
0.10		0.710	
0.20		0.961	
0.25		0.997	
0.30		1.015	
0.35		1.024	
0.40		1.032	
0.45		1.038	
0.50	1.048	1.038	1.010
0.55		1.034	
0.60	1.041	1.032	1.009
0.65		1.032	
0.70	1.042	1.026	1.016
0.75		1.024	
0.80	1.027	1.018	1.009
0.85		1.016	
0.90	1.013	1.008	1.005
0.95		0.999	
1.00	1.000	1.000	1.000
1.20		0.976	
1.30		0.957	
1.50	0.923	0.931	0.991
2.00	0.834	0.851	0.980
2.50	0.750	0.769	0.975
3.00	0.669	0.688	0.972
3.50	0.592	0.612	0.967
4.00	0.523	0.541	0.967
4.50		0.475	
5.00	0.399	0.417	0.957
5.50		0.367	
6.00	0.305	0.318	0.959
7.00	0.222	0.242	0.917
8.00	0.163	0.183	0.891
9.00	0.126	0.136	0.926
10.00	0.090	0.102	0.882

**Table 2:** Cs‐1 radial dose functions obtained by this work compared with a previous study by Wang and Zhang^(^ ^20^ ^)^ in a water phantom.

	*g*_*L*_ *(r)*	*g*_*L*_ *(r)*	*Ratio*
*Z (cm)*	*A. Wang et al.* ^(^ ^20^ ^)^ *(MCNP5)*	*B. This work, 30 cm in diameter spherical water phantom, torus tally cells. (MCNPX)*	A/B
0.10		0.961	
0.20	0.981	0.967	1.014
0.25	0.988	0.975	1.013
0.30	0.998	0.979	1.019
0.35		0.987	
0.40	1.003	0.988	1.015
0.45		0.997	
0.50	1.007	0.997	1.010
0.55		0.999	
0.60	1.006	1.001	1.005
0.65		1.003	
0.70	1.008	1.001	1.007
0.75		1.005	
0.80	1.007	1.001	1.006
0.85		1.000	
0.90	1.004	1.002	1.002
0.95		1.002	
1.00	1.000	1.000	1.000
1.20		0.991	
1.30		0.989	
1.50	0.963	0.978	0.985
2.00	0.909	0.934	0.973
2.50	0.846	0.880	0.961
3.00	0.778	0.818	0.951
3.50		0.755	
4.00	0.641	0.691	0.928
4.50		0.630	
5.00	0.516	0.571	0.904
5.50		0.515	
6.00	0.410	0.463	0.886
7.00	0.321	0.370	0.868
8.00	0.250	0.293	0.853
9.00		0.230	
10.00	0.147	0.179	0.821

### B. Source strengths in two types of eye plaques

Our results indicate that, to achieve a prescription dose of 85 Gy at 6 mm from the inner sclera edge, the OSU‐NAG eye plaque will need a source strength of 6.16 U/source (49.28 U in total) and 6.82 U/source (54.56 U in total) for IAI‐125A and Cs‐1 sources, respectively, but the COMS eye plaque will require 4.02 U/source (52.26 U in total) and 4.43 U/source (57.59 U in total) for the same source types for a six‐day treatment. When the data by Melhus et al.^(^ ^11^ ^)^ for 16 mm COMS eye plaque were normalized to a 6 mm prescription point (originally at 5 mm) and corrected to our actual treatment time of six days from the seven days used in the original study, we found the air kema strength per source obtained from our 16 mm COMS calculation to be 13% different from Melhus' for ${\;}^{131}\text{Cs}$ source (Table 3).

**Table 3:** Dose comparison between of the OSU‐NAG and COMS eye plaques. Results by Melhus^(^ ^11^ ^)^ were normalized to the dose at 6 mm and corrected for the treatment time used in this study (6 days vs. 7 days in the Melhus study).

	*IAI‐125A I‐125*				*CS‐1 Cs‐131*		
*Z (cm)*	*OSU‐NAG (Gy)*	*COMS (Gy)*	*Ratio (OSU‐NAG/COMS)*	*OSU‐NAG (Gy)*	*COMS (Gy)*	*COMS‐Melhus (Cs‐1 Cs‐131) (Gy)*	*Ratio (OSU‐NAG/COMS)*
‐0.1	437.5	415.3	1.05	398.7	365.0	352.0	1.09
0.0	356.2	323.0	1.10	331.3	294.6	293.8	1.12
0.1	278.6	256.3	1.09	261.9	236.7	236.7	1.11
0.2	215.5	200.9	1.07	205.6	190.8	191.5	1.08
0.3	167.2	162.3	1.03	162.9	154.5	155.8	1.05
0.4	131.9	129.0	1.02	129.4	126.0	126.7	1.03
0.5	104.8	104.5	1.00	104.0	102.6	103.1	1.01
0.6	85.0	85.0	1.00	85.0	85.0	85.0	1.00
0.7	68.3	70.8	0.96	69.9	70.9	71.1	0.99
0.8	56.6	59.4	0.95	58.2	59.6	59.3	0.98
0.9	47.3	50.3	0.94	48.3	50.0	50.0	0.97
1.0	39.9	42.4	0.94	41.2	42.8	42.6	0.96
1.2	28.9	30.6	0.95	30.7	31.8		0.97
1.4	21.5	22.9	0.94	23.3	24.1		0.97
1.6	16.8	17.7	0.95	18.0	18.6		0.97
1.8	13.0	13.5	0.96	14.3	14.7		0.97
2.0	10.3	10.3	0.99	11.5	12.0		0.96
2.2	8.2	8.4	0.97	9.5	9.7		0.98
2.4	6.5	6.9	0.94	7.8	8.0		0.97
2.6	5.5	5.7	0.96	6.5	6.6		0.99
2.8	4.4	4.8	0.93	5.2	5.6		0.93
3.0	3.7	4.0	0.92	4.5	4.9		0.92
*Sk* (U/source)	6.16	4.02		6.82	4.43	5.03	

### C. Doses along the central axis of eye plaque

The doses along the eye plaque axis are shown in Table 3 which lists the doses and their ratios at the central axis from two types of eye plaques loaded with two types of brachytherapy sources separately, and the doses derived from the Melhus study.^(^ ^11^ ^)^ Our results indicate that the doses from the two plaque types were comparable on the central axis. The 16 mm OSU‐NAG plaque will give a slightly larger dose than the COMS at short distances (smaller than 6 mm). At the inner sclera edge (Z = 0), the dose from the OSU‐NAG plaque would be 10% and 12% larger than that for the COMS eye plaque, respectively, for I‐125 and Cs‐131 sources. At 24 mm (this point is in the healthy eyeball and the opposite of retina), the OSU‐NAG dose would be 6% and 3% smaller than that of COMS. Since the shorter distance region is in the tumor and the large distance is in the healthy part of eyeball, the dosimetric situation generated by the OSU‐NAG plaque would be clinically unremarkable. Our results agree with the Melhus study within 4% and 0.1%, respectively, for the IAI‐125A (6711 source was used in the Melhus study) and Cs‐1 sources, except at Z = 0 and −1.0 mm.

### D. Dose volume histogram (DVH) of tumor

**Figure 5:**
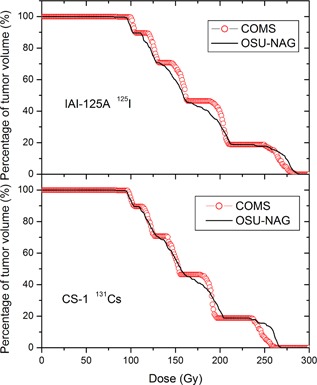
Tumor dose‐volume histogram comparison between the 16 mm SU‐NAG and 16 mm COMS eye plaques loaded with I‐125 and Cs‐131 sources separately.

Figure 5 shows the comparisons of tumor DVHs from two types of eye plaques at the same prescription dose of 85 Gy for two types of sources. The DVHs of the tumor show a minor difference between two eye plaque types; the use of the OSU‐NAG plaque creates slightly hotter dose regions than the COMS (longer tail of DVH curve). V120% is the same for both types of eye plaques, V150% of the COMS is 5% higher than that seen from the OSU‐NAG.

## IV. DISCUSSION

In this study, OSU‐NAG eye plaque brachytherapy has been compared with the COMS eye plaque using ${\;}^{125}I$ and ${\;}^{131}\text{Cs}$ brachytherapy sources. In order to have a fair comparison, the Silastic source carrier of the COMS eye plaque was replaced by a water source carrier which does not attenuate the dose and, thus, does not need to be modeled in treatment planning systems. In this study, we have also avoided the uncertainty of compositions and geometry in Silastic source carrier. The results of this study indicated that the 16 mm OSU‐NAG eye plaque has similar dosimetric characteristics compared with the 16 mm COMS eye plaque using either the ${\;}^{125}I$ or ${\;}^{131}\text{Cs}$ sources.

A comparison between the current study and the Melhus study demonstrates that the Silastic carrier does have a dosimetric impact. However, the final doses after attenuation correction are close to those used in our study, which does not use the Silastic carrier except at the points very close to the plaque (z = −1 mm, 0). The Silastic carrier would significantly increase (13% ~ 15% in this case) the required source strength necessary to deliver the prescription dose.

Applying the general concepts, one would expect to see hotter dose regions when fewer sources are used. This is confirmed in our study in which the OSU‐NAG eye plaque was found to have hotter dose regions than COMS eye plaque (Fig. 5). Surprisingly, the OSU‐NAG eye plaque was found to create only slightly hotter dose regions than COMS eye plaque. In the COMS eye plaque, the maximum dose, D 0.1 cc for example, is less than that in OSU‐NAG eye plaque. D 0.1 cc is 274 Gy for ${\;}^{125}I$, and 256 Gy for ${\;}^{131}\text{Cs}$, while the values in the OSU‐NAG eye plaque are 281 Gy and 264 Gy, respectively. This implies that a moderate number of sources might be enough to maintain adequate dose coverage while avoiding a significant increase of hot dose regions. It should be noted that this also could be achieved by using fewer seeds in the COMS eye plaques.

In addition, the tumor DVH indicates that the COMS plaque has a 3% larger high‐dose volume than the OSU‐NAG eye plaque at some high‐dose regions (greater than 85 Gy). Using ${\;}^{125}I$ sources, V125, V150, and V200 in COMS eye plaque are 89.6%, 76.5%, and 46.4%; the corresponding values in OSU‐NAG eye plaque are 89.6%, 71.9%, and 44.5%, respectively. This is because brachytherapy sources generally create hot dose zones at the vicinity of the sources; thus, using more sources would create more high‐dose volumes. Our clinical observations in over 119 cases,^(^ ^27^ ^)^ where OSU‐NAG eye plaques were utilized, demonstrated that the clinical outcomes and complications were comparable to those reported for the COMS eye plaques.^(^ ^28^ ^)^

Currently, most clinical data involving eye plaque brachytherapy use ${\;}^{125}I$ and ${\;}^{103}\text{Pd}$ sources, and other sources are not widely used. The radiobiology implication introduced by different sources has not been systematically investigated by experimental or clinical trials. ${\;}^{131}\text{Cs}$ has a much shorter half‐life than ${\;}^{125}I$ and ${\;}^{103}\text{Pd}$. Therefore, it would deliver an effective dose in a shorter period of time, and require hotter sources than ${\;}^{125}I$ and ${\;}^{103}\text{Pd}$. In addition, the disadvantage of a shorter half‐life is that the operating room time has to be more tightly scheduled to deliver the radiotherapy (e.g., significantly less/more dose would be delivered if the plaque is removed early or late due to an unforeseen emergency situation). So far, the clinical use of ${\;}^{131}\text{Cs}$ sources in eye plaque brachytherapy has not been reported. Since the initial dose rate of ${\;}^{131}\text{Cs}$ source is ~ 40% larger than ${\;}^{125}I$ sources, the biology effect of ${\;}^{131}\text{Cs}$ eye plaque brachytherapy may be quite different. A more comprehensive study involving biological effects to reveal the pros and cons of all types of sources is expected.

## V. CONCLUSIONS

Compared with the fully loaded 16 mm COMS eye plaque, the 16 mm OSU‐NAG eye plaque can achieve similar dosimetric characteristics with fewer sources. Therefore, from a dosimetric perspective, the OSU‐Nag plaque can achieve similar results and is an alternative to the COMS eye plaque (as are the USC and ROPES plaques).

## ACKNOWLEDGMENTS

The author HZ thanks Dr. Sou‐Tong Chiu‐Tsao, AAPM eye plaque task group (TG‐129) chair, for providing the valuable consultation during the study. Authors also acknowledge Dr. Subir Nag for helpful discussion of his initial idea of four‐seed Nag eye plaques. It should be noted that the current eight‐seed OSU eye plaques were evolved from Nag's initial idea. The authors also would like to thank Deborah MacDougall of Indiana University for proofreading the manuscript.
